# A Novel *CDC42* Mutation in an 11-Year Old Child Manifesting as Syndromic Immunodeficiency, Autoinflammation, Hemophagocytic Lymphohistiocytosis, and Malignancy: A Case Report

**DOI:** 10.3389/fimmu.2020.00318

**Published:** 2020-03-13

**Authors:** Aleksandra Szczawinska-Poplonyk, Rafal Ploski, Ewa Bernatowska, Malgorzata Pac

**Affiliations:** ^1^Department of Pediatric Pneumonology, Allergology and Clinical Immunology, Poznan University of Medical Sciences, Poznan, Poland; ^2^Department of Medical Genetics, Medical University of Warsaw, Warsaw, Poland; ^3^Department of Immunology, Children's Memorial Health Institute, Warsaw, Poland

**Keywords:** Cdc42, immunodeficiency, hemophagocitic lymphohistiocytosis, malignancy, gene mutation

## Abstract

**Background:** The *CDC42* (*Cell Division Cycle 42*) gene product, CDC42, is a member of the family of small Rho GTPases, which are implicated in a broad spectrum of physiological functions in cell cycle regulation, including establishing and controlling of the cell actin cytoskeleton, vesicle trafficking, cell polarity, proliferation, motility and migration, transcription activation, reactive oxygen species production, and tumorigenesis. The *CDC42* gene mutations are associated with distinct clinical phenotypes characterized by neurodevelopmental, growth, hematological, and immunological disturbances.

**Case presentation:** We report the case of an 11-year-old boy with syndromic features, immunodeficiency, and autoinflammation who developed hemophagocytic lymphohistiocytosis and malignant lymphoproliferation. In this patient, a novel heterozygous p.Cys81Tyr mutation in the *CDC42* gene was found by whole exome sequencing.

**Conclusions:** The Cdc42 molecule plays a pivotal role in cell cycle regulation and a wide array of tissue-specific functions, and its deregulation may result in a broad spectrum of molecular and cellular dysfunctions, making patients with *CDC42* gene mutations susceptible to infections, immune dysregulation, and malignancy. In the patient studied, a syndromic phenotype with facial dysmorphism, neurodevelopmental delay, immunodeficiency, autoinflammation, and hemophagocytic lymphohistiocytosis shares common features with Takenouchi–Kosaki syndrome and with C-terminal variants in *CDC42*. It is important to emphasize that Hodgkin's lymphoma is described for the first time in the medical literature in a pediatric patient with the novel p.Cys81Tyr mutation in the *CDC42* gene. Further studies are required to delineate precisely the *CDC42* genotype–phenotype correlations.

## Background

The *CDC42* (*Cell Division Cycle 42*) gene product, CDC42, is a member of the family of Rho GTPases (small G proteins of the Rho-family), which belongs to the Ras superfamily of small GTPases. The Rho-family GTPases have a broad spectrum of physiological functions in cell cycle regulation, including establishing and controlling of the cell actin cytoskeleton, vesicle trafficking, cell polarity, cell proliferation, motility and migration, transcription activation, reactive oxygen species production, and tumorigenesis (1). The signaling and the regulatory function of CDC42 are based on its tightly regulated cycle of activation with GTP binding and an inactive state with GTP hydrolysis, and intricate interactions with multiple proteins that impact on cell functions during its active state (2). It has been shown that CDC42 plays a number of physiologically pivotal, tissue-specific roles in the cardiovascular, genitourinary, respiratory, nervous, and immune systems, and its dysfunction is implicated as a background for syndromic immunodeficiency and immune dysregulation in patients reported so far (3–9). This is the first report of a pediatric patient with syndromic immunodeficiency, autoinflammation, hemophagocytic lymphohistiocytosis, and malignant lymphoproliferation in whom a novel, heterozygous p.Cys81Tyr mutation in the *CDC42* gene was found.

## Case Presentation

We report the case of a 9-year-old boy who was referred to the pneumonology, allergology, and clinical immunology unit of the Poznan Pediatric University Hospital because of pneumonia, bilateral otitis media, and vesicular dermatitis.

Since the age of 2 years, he suffered from recurrent respiratory tract infections and required multiple hospitalizations because of recurrent bronchitis and pneumonia, maxillary sinusitis, otitis media, purulent dermatitis with *Pseudomonas aeruginosa* and *Staphylococcus aureus* infection, and severe varicella complicated by pneumonia, sinusitis, and gastrointestinal infection. He completed a full course of vaccinations, including BCG (Bacille Calmette–Guerin) and MMR (measles–mumps–rubella) vaccines without adverse effects following immunization (AEFI). The family history was complicated by multiple sclerosis in the patient's father.

He presented with neurodevelopmental delay and dysmorphic features with oblique palpebral fissures and eyebrows, retrognathia, low set small auricles with thick helices, and clinodactyly of the V fingers. The erythematous papulovesicular rash was present on the skin of the face, in the perioral region, and in the retroauricular area. In the nasopharynx and in the oral cavity, inflammatory lesions were observed. The most striking symptom was lymphadenopathy with numerous bilaterally enlarged cervical and submandibular lymph nodes. During hospitalization, he required antibiotic therapy, bilateral paracenthesis with tympanostomy, and drainage of maxillary sinuses. Laboratory evaluation revealed an antibody production defect and a memory B cell deficiency. Therefore, replacement therapy with intravenous immunoglobulin (IVIg) was initiated, and further genetic testing was recommended. The patient received three IVIg transfusions in monthly intervals, but afterward the parents decided to discontinue the therapy, and the boy was lost to follow-up.

At the age of 11 years, the boy was referred again to our clinic because of recurrent fevers, accompanied by vomiting, abdominal pain, cervical lymphadenopathy, and splenomegaly. The episodes of fever started 5 months before the hospitalization; they were not associated with any signs and symptoms of infection, they would reach 39.5 degrees, and did not respond to treatment with antibiotics. At that time, no other family members were ill, the boy had no contact with any toxic substances or infections, and he did not travel to the Mediterranean or exotic regions.

The laboratory tests showed pancytopenia, lymphopenia and neutropenia, high inflammatory markers, hypoalbuminemia, IgG and IgM hypoimmunoglobulinemia, hyperferritinemia, hypertriglyceridemia, hypertransaminasemia, and positive EBV-DNA (93,400 copies/ml) in the peripheral blood. Further laboratory findings comprised a markedly elevated (7,255 U/ml) serum concentration of the soluble interleukin 2 receptor (sIL-2R, sCD25) and a decreased intracellular expression of perforin (CD107a) on NK cells and increased on CD8+ T cells. Concomitantly, neither in the bone marrow nor in the lymph node were signs of hemophagocytosis found. Hence, seven of the eight diagnostic criteria (four clinical and three immunological criteria) of hemophagocytic lymphohistiocytosis (HLH) were fulfilled (10) (data displayed in Table 1).

**Table 1:** Results of laboratory investigations in the patient studied aged 11 years.

**Test**	**Results**
Immunology	•Pancytopenia WBC 1.68 × 10^3^, HGB 8.9 G/L, RBC 3,10 × 10^6^, PLT 39 × 10^3^ •Hypoimmunoglobulinemia IgG 479 mg/dl, IgM 11 mg/dl, IgA < 5 mg/dl •Peripheral blood lymphocyte immunophenotyping Lymphocytes CD45+/SSC low: 38% (1,125/mcl) low T CD3+ 81.0% (930 cc), low Th CD4+ 17.0% (195/mcl), high Tc CD8+ 59.0% (677/mcl) markedly decreased CD4+/CD8+ ratio 0.29, very low B cells CD19+ 6% (69/mcl) NK CD3–CD45+CD16+CD56+ 9.0% (103/mcl), activated CD3+HLA-DR+ 58% Low Th naïve CD4+CD45RA+ 10.0% (20/mcl), Th memory CD4+CD45RO+ 90.0% (176/mcl) low CD4+CD45RA+/CD4+CD45RO+ ratio 0.11 Low Recent thymic emigrants CD4+CD31+CD45RA+ 9.0% (18/mcl) Low Th Naïve CD4+CD27+CD45RO– 14.7% (29/mcl) Th Central memory CD4+CD27+CD45RO+ 72.8% high (142/mcl) low Low Th Effector memory CD4+CD27-CD45RO+ 10.6% (21/mcl) Low Th Terminally differentiated memory CD4+CD27-CD45RO– 1.8% (4/mcl) Th Regulatory CD4+CD127-CD25+ 6.7% (13/mcl) T follicular helper CD4+CD45RO+CD185+ 35.1% (62/mcl) Markedly decreased Tc Naïve CD8+CD27+CD197+ 10.2% (69/mcl) Tc Central memory CD8+CD27+CD45RO+ 32.9% (223/mcl) high Tc Effector memory CD8+CD27–CD197 × CD45RO+ 53.7% (364/mcl) CD107a decreased intracellular expression on NK cells, increased on CD8+ T cells
Microbiology	•CMV-DNA positive •EBV-DNA I. 12,900 copies/ml–II. 93,400 copies/ml •VZV, Enterovirus, Adenovirus, Parechovirus, HBV, HCV, HIV, hPVB19, HSV1, HSV2, HHV6, HHV7 RT-PCR in peripheral blood negative •Influenza virus A, AH1N1, Influenza virus B, Coronavirus NL63, 229E, OC43, HKU1, Parainfluenzavirus 1,2,3,4, Metapneumovirus A,B, Bocavirus, RSV A,B, Rhinovirus, Adenovirus, Enterovirus, Parechovirus, *Mycoplasma pneumoniae, Chlamydophila pneumoniae, Streptococcus pneumoniae, Staphylococcus aureus* RT-PCR in nasopharyngeal aspirate negative •*Yersinia enterocolitica* IgM, IgG, IgA negative •*Bartonella henselae* DNA negative •*Toxoplasma gondii* DNA negative •Galactomannan (Aspergillus antigen) negative •*Pneumocystis jiroveci* DNA negative •Quantiferon TB Gold negative •Blood, throat, urine cultures negative
Inflammatory markers	•CRP 22.52 mg/dl •PCT 3.90 ng/ml •Ferritin 3,649.4 ng/ml, TG 365.0 mg/dl, Fibrinogen 504 mg/dl •sIL-2R (sCD25) 7,255 U/ml
Neoplastic markers	•Beta-HCG <2.39 mIU/ml •AFP 1.5 ng/ml •LDH 124 IU/L
Histopathology	•Supraclavicular (Virchow's) lymph node: classical Hodgkin's lymphoma, nodular sclerosis •Immunohistochemistry: large atypical lymphoma cells positive for CD30, CD15, PAX-5, MUM.1, LMP/EBV, EBI-3, granzyme B, EMA; negative for ALK-1, CD43, CD3, CD20

**Figure 1:**
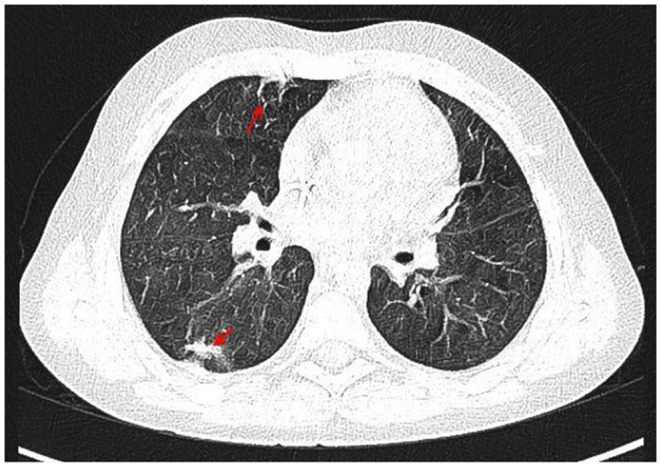
CT of the chest with contrast medium, arterial phase, coronal view. Irregular, thickened peribronchium with narrowing of the lumen of segmental and subsegmental bronchi, fibrosis (marked with arrows) with compensatory dilation of the bronchial lumen (bronchiectasis), signs of bronchiolitis in PS10 and LS 10, irregular nodules in LS9 and LS10, a trace of fluid in the pericardium, enlarged right upper paratracheal, below carina, paraaortic, perivascular lymph nodes.

**Figure 2:**
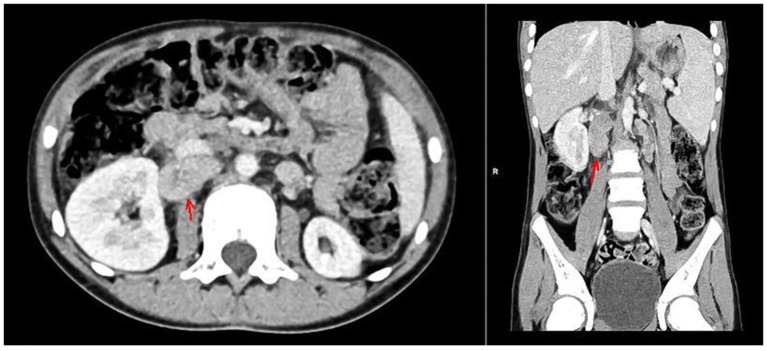
CT of the abdomen with contrast medium, arterial phase, axial, and coronal views. Hypodense foci in the II and VIII segments of the liver, enlarged spleen, *ca*. 14 cm long with nonhomogenous attenuation, hypoplastic left kidney, lymphadenopathy of lymph nodes in the regions of the head of the pancreas, portal vein, right renal hilum, inferior vena cava (marked with arrows), and retroperitoneal, iliac and aortic lymph nodes, forming a mass 8.5 cm long.

With the concern of severe respiratory infection (Figure 1) and sepsis in an immunocompromised patient, initial empiric pharmacotherapy was based on broad spectrum antibiotics meropenem and vancomycin, *Pneumocystis jiroveci* prophylaxis with cotrimoxazole, and antiviral and antimycotic medications acyclovir and fluconazole. The chemo-immunotherapy for HLH was initiated with methylprednisolone pulse therapy, etoposide, and cyclosporine. The boy also required supplemental transfusions of albumins, immunoglobulins, prothrombin complex, and red blood cell preparations. The initial response to the therapy was satisfactory with an improvement in the patient's general state, a resolution of fevers, and a decrease in the serum inflammatory markers. Subsequently, however, the boy's state deteriorated, febrile episodes returned, and exacerbation of the supraclavicular and abdominal lymphadenopathy was observed (Figure 2). Based on complex diagnostic procedures including histopathology, immunology, and magnetic resonance imaging (MRI), the diagnosis of IV stage nodular sclerosis (NS) Hodgkin's lymphoma was established. The boy underwent successful chemotherapy based on the Euronet Pediatric Hodgkin's Lymphoma protocol (EuroNet-PHL-C1) including OEPA (vincristine, etoposide, prednisone, and doxorubicin) followed by 18-fluorodeoxyglucose-positron emission tomography-computed tomography (FDG-PET-CT) reevaluation. Further considerations and decisions regarding therapeutic options, including bone marrow transplantation, are ongoing.

## Genetic Analysis

In the search for the genetic cause of HLH, syndromic immune deficiency, autoinflammation, and recurrent fevers, whole exome sequencing (WES) of DNA extracted from peripheral blood was performed. For the enrichment SureSelectXT Human All Exon v7 (Agilent) was used and sequencing was performed on the Illumina Platform HiSeq 1500. Bioinformatics analysis was performed as previously described with the modification that the Hg38 version of the human genome reference sequence was used for alignment (11). The details of the WES results and variant filtering are given in the Supplementary Material.

We prioritized a heterozygous missense variant in the *CDC42* gene (NM_044472.3 c.242G>A, p.Cys81Tyr, Hg38:1:022086502-G>A, LRG_1326t2:c.[242G>A];[242=]). The variant has not been reported previously; it was not found in the GnomAD database (https://gnomad.broadinstitute.org) nor in an in-house database of >1,000 Polish exomes. The variant was predicted to be pathogenic by Mutation Taster, Mutation Assessor, FATHMM-MKL, FATHMM-XF, LRT, DEOGEN2, EIGEN, EIGEN PC, SIFT, SIFT4G, PROVEAN, MVP, REVEL, PrimateAI, MetaSVM, and MetaLR, whereas FATHMM suggested that it was “tolerated” (https://varsome.com). The CADD score (28,1) indicated pathogenicity. Notably, an alternative variant at the same amino acid position (chr1:22086502 G>T, Cys81Phe) has been classified as “Likely Pathogenic” in association with the Takenouchi–Kosaki syndrome by ClinVar (https://www.ncbi.nlm.nih.gov/clinvar/). The fragment of the *CDC42* gene encompassing the c.242G>A variant was PCR-amplified and analyzed with the method of deep amplicon sequencing (ADS) in the proband and his parents. The presence of the variant was confirmed in the proband and excluded in both parents, indicating that the variant occurred *de novo*.

According to the ACMG recommendations (12), the variant was classified as “Likely Pathogenic.” This was based on the following criteria: the variant resulted from a *de novo* mutation in a patient with no family history (Pathogenic Strong, PS2); the variant not found in GnomAD data (Pathogenic Moderate, PM2); an alternative variant at the same amino acid position classified as Likely Pathogenic by ClinVar (Pathogenic Moderate, PM5); multiple lines of computational evidence supporting a deleterious effect (Pathogenic Supporting, PP3).

## Discussion

The broad spectrum of heterogeneous clinical manifestations in disturbed CDC42 molecule regulation results from the wide variety of cellular pathways with CDC42 activity. In a mammalian model, it has been shown that CDC42 plays a fundamental role in cell biology and is implicated in a wide array of physiologically pivotal, tissue-specific activities, such as organogenesis, cellular specification, migration, and functional maturation in the cardiovascular system, the pancreas, the kidney, the lung, salivary and mammary glands, the central nervous system (2, 13), the skin (2), the bones (2), the sensory organ of the inner ear (1, 14), and the photoreceptors (15). An important clinicopathological issue is the role of CDC42 GTPase in hematopoiesis and in immune system homeostasis. CDC42 controls the multilineage development of blood progenitors and their egress from the bone marrow to the periphery, influences the tight balance between myelopoiesis and erythropoiesis (16, 17), and contributes to the immune system regulation by coordinating survival, proliferation, migration, receptor expression, activation signal transduction, and cytokine secretion in T (18–20) and B cells (21). Dysregulated CDC42-dependent actin dynamics may, therefore, impede multiple stages of B cell development and affinity maturation, resulting in an aberrant germinal center response and immunodeficiency, autoimmunity, and lymphoproliferation (22).

In the patient studied, in whom a novel, heterozygous *de novo* p.Cys81Tyr mutation in the *CDC42* gene was identified for the first time, a marked complexity of the clinical phenotype is observable, characterized by syndromic features, neurodevelopmental delay, immunodeficiency, autoinflammation, hemophagocytic lymphohistiocytosis, and malignant lymphoproliferation. The clinical phenotype of patients with a *CDC42* gene mutation and the triad of recognizable symptoms, such as macrothrombocytopenia, developmental delay, and distinctive facial features, has been initially described by Takenouchi and Kosaki in 2015 (3). It is worth noting that the initially reported patient with the Takenouchi–Kosaki syndrome and the p.Tyr64Cys mutation did not present with the diverseness of clinical manifestations observable in our patient with the p.Cys81Tyr variant since immunodeficiency, systemic autoinflammatory disease, HLH, and lymphoma were not present in the original case. Further evidence supporting the notion that the *CDC42* gene mutation causes a syndromic form of thrombocytopenia has also been provided by Takenouchi et al. (4) and Motokawa et al. (5) in their reports of the next two patients, sharing the same p.Tyr64Cys variant (6) and overlapping phenotypes including facial dysmorphism, psychomotor developmental delay, lymphedema of the lower extremities, camptodactyly, sensorineural hearing loss, and immunodeficiency. Further evidence for the intricate genotype–phenotype relationship and the heterogeneity of the clinical features correlating with mutations affecting the *CDC42* gene was provided by Martinelli et al. (7). The authors reported 15 patients, divided in three groups according to different disease-causing *CDC42* mutations, namely, group I—p.Tyr64Cys, p.Arg66Gly, p.Arg68Gln, group II—p.Cys81Phe, p.Ser83Pro, p.Ala159Val, and group III—p.Ile21Thr, p.Tyr23Cys, pGlu171Lys. The various mutations are related to an unusually broad spectrum of anomalies with predominant growth retardation and developmental disorders with an intellectual disability, while macrothrombocytopenia was noted foremostly in group I. In patients with the p.Cys81Phe variant, affecting the same amino acid as in the novel p.Cys81Tyr here reported, severe autoinflammatory disease, lymphohistiocytic hemophagocytosis, and lymphoproliferation were not present. In contrast to patients with the classical Takenouchi–Kosaki syndrome and similarly to our patient, systemic autoinflammatory disease and development of HLH were predominating manifestations in four patients with three distinct C-terminal *de novo* variants (p.C188Y, p.R186C, p.^*^192C^*^24) in *CDC42* reported by Gernez et al. (8). Likewise, four patients with disturbed hematopoiesis, rash, autoinflammation, and HLH, reported by Lam et al. (9), were sharing the same C-terminal p.R186C variant in the *CDC42* gene. The two latter studies point to the unique effect of the C-terminal mutations in *CDC42*, resulting in neonatal-onset deregulation of the inflammatory response and the development of secondary HLH. While our patient did not present with symptoms of a significant autoinflammatory disease and HLH in his early childhood, it may be assumed that HLH might be secondary in the setting of autoinflammation and malignant transformation.

Since CDC42 plays a pivotal role in a tight regulation of a plethora of complex cell functions, its deregulation resulting in impaired cell proliferation, migration, and transcription programming, was shown to be oncogenic. Overexpression of CDC42 was reported in several cancers, including non-small cell lung cancer, colorectal adenocarcinoma, melanoma, breast cancer, and testicular cancer, supporting the role of CDC42 in the promotion of tumorigenesis as an oncogene (23). The modulation of transcription factors, such as signal transducer and activator of transcription 3 (STAT3) and nuclear factor κB (NFκB), which, in turn, regulate cancer cell growth and survival, as well as alter cancer cell metabolism (24), is a further mechanism by which the activation of CDC42 contributes to malignant cell transformation. Numerous, somatic genetic alterations affecting CDC42 were described in human lymphomas (25). While the *CDC42* mutation may play a role in the genesis of the lymphoma, it may be assumed that hematopoietic stem cell transplantation would be a curative strategy for the patient. However, whether the development of Hodgkin's lymphoma in the reported patient may be mechanistically connected with the novel p.Cys81Tyr variant in the *CDC42* requires further investigations.

## Concluding Remarks

We describe a novel heterozygous p.Cys81Tyr mutation in the *CDC42* gene, which encodes a small GTPase of the Rho subfamily, playing a pivotal role in cell cycle regulation. In the patient studied, diverse clinical manifestations compose a syndromic phenotype with facial dysmorphism, neurodevelopmental delay, immunodeficiency, autoinflammation, hemophagocytic lymphohistiocytosis, and malignant lymphoproliferation, sharing common features with the Takenouchi–Kosaki syndrome and patients with the C-terminal *CDC42* mutations. It is worth noting that Hodgkin's lymphoma is described here for the first time in a patient with a mutation in *CDC42*. It may be therefore assumed that different disease-causing *CDC42* mutations have a different impact on the GTPase structure, activity, and binding to effectors, and finally, on the heterogeneity of clinical manifestations in affected patients, leading to new challenges in the syndrome's recognition and delineation.

## Data Availability Statement

The raw data supporting the conclusions of this article will be made available by the authors, without undue reservation, to any qualified researcher.

## Ethics Statement

Ethical review and approval was not required for the study on human participants in accordance with the local legislation and institutional requirements. Written informed consent to participate in this study was provided by the participants' legal guardian/next of kin. Written informed consent was obtained from the parent for the publication of this case report.

## Author Contributions

AS-P was responsible for the conception and design of the study, collection and interpretation of clinical data, and drafted the manuscript. RP was responsible for genetic analysis and interpretation of data and critically revised the manuscript. MP and EB participated in the clinical evaluation of the case and critically revised the manuscript.

### Conflict of Interest

The authors declare that the research was conducted in the absence of any commercial or financial relationships that could be construed as a potential conflict of interest.
