# Supplementary material for: A Novel CDC42 Mutation in an 11-Year Old Child Manifesting as Syndromic Immunodeficiency, Autoinflammation, Hemophagocytic Lymphohistiocytosis, and Malignancy: A Case Report
Source: Front Immunol. 2020 Mar 13;11:318. doi: 10.3389/fimmu.2020.00318 (PMC7082228; doi:10.3389/fimmu.2020.00318)
Supplement: Supplementary file 1 [file Data_Sheet_1.pdf]

Whole exome sequencing and variant filtering

During sequencing 96 959 840 reads were generated. The mean coverage of the target was 99,1; 98% of the target was covered min 10x; 95% -min 20x.

In total there were 123 805 variants identified which passed the default quality filter.

The variants were filtered to include those with low frequency and a predicted effect on the protein. The frequency filter removed variants with prevalence >1% in GnomAD or a Polish database of >1000 WES. The predicted effect filter excluded, synonymous and non-coding variants unless they were located within 20 bp from the end of an exon). Variants annotated in ClinVar as Pathogenic/Likely pathogenic were retained regardless of their frequency/function.

After the filtering here were 526 variants left. These were manually inspected Using Integrative Genomics Viewer (IVG) and considered by an expert regarding available knowledge on gene function and its disease associations. The variants found in the proband's sample that were considered as potentially disease-causing in addition to *CDC42* c.[242G>A] are shown in Supplemental Table 1.

Supplemental Table 1. Variants found in the proband's sample that were considered as potentially disease-causing in addition to *CDC42* c.[242G>A].

Gene	Position (Hg38)	ID	Effect	gnomAD freq.	Disease (inheritance) / comments
**Variants listed as pathogenic/likely pathogenic by ClinVar**					
ZC3H14	14:88610949-del TT…AG(25bp)	rs571303442	NM_024824.5:c.2204+17_2204+41del	0,011	Mental retardation,56, MIM# 617125 (AR)/ heterozygous variant, no other variant in the same gene, not consistent with probands's symptoms.
PPA2	4:105437964-C>T	rs146013446	NP_789845.1:p.Glu172Lys	0,0007	Sudden cardiac failure, alcohol-induced, MIM# 617223, (AR)/ heterozygous variant, no other variant in the same gene, not consistent with probands's symptoms.
ABCA4	1:094008251-C>T	rs1800553	NP_000341.2:p.Gly1961Glu	0,003	Stargardt disease 1,MIM# 248200, (AR)/ heterozygous variant, no other variant in the same gene, phenotype not consistent with probands's symptoms.
**Potentially biallelic variants with freq.<0,01 in GnomAD and an in-house Polish database with min. 1 variant predicted as pathogenic by >5 out of 9 prediction software tools used #**					
PKHD1	6:52060028-C>T	rs1311172284	NP_619639.3:p.Gly378Glu	0,00001	Polycystic kidney disease 4, MIM# 263200 (AR)/ not consistent with probands's symptoms.
	6:51883179-A>C	rs201881567	NP_619639.3:p.Cys2422Gly	0,0007	
ADAMTS7	15:78790696-G>C	rs150760240	NP_055087.2:p.His334Gln	0,0005	No disease association in human, mouse knock-out without immunological phenotype (www.informatics.jax.org)
	15:78766345-G>T	rs139513941	NP_055087.2:p.Thr1189Asn	0,0006	
MMP1	11:102797320-C>T	rs148980271	NP_002412.1:p.Asp96Asn	0,0010	COPD, rate of decline of lung function in, MIM#606963 (AR); Epidermolysis bullosa dystrophica, autosomal recessive, modifier of, MIM# 226600 (AR)/ not consistent with probands's symptoms.
	11:102796715-CA>C	rs17879749	NP_002412.1:p.Ile191MetfsTer45	0,0113	
**Potentially AD/XLD/XLR (predicted to cause LoF and/or predicted as pathogenic by MetaSVM, freq.=0 in all available databases)**					
RACGAP1	12:050001172-C>T		Synonymous variant predicted to affect splicing (ADA score >0,999)	0	No known disease association, predicted to tolerate monoallelic LoF (pLI= 0,002)
DHCR7	11:071435785-C>A		NP_001351.2:p.Val340Phe	0	Pathogenic *DHCR7* variants cause Smith-Lemli-Opitz syndrome, MIM#270400, AR inheritance
PCDHA11	5:140871003-C>T		NP_061725.1:p.Arg634Cys	0	No known disease association, predicted to tolerate biallelic LoF (pNull=0,95)
DNAH11	7:021588578-CA>TG		NP_001264044.1:p.Gln639Trp		Pathogenic *DNAH11* variants cause Primary ciliary dyskinesia 7, MIM# 611884, AR inheritance.

AR – autosomal recessive, AD – autosomal dominant, XLD/R – X linked dominant/recessive, MIM-Mendelian inheritance in man * pLI = probability of Loss of Function (LoF); the closer pLI is to one, the more LoF intolerant the gene to monoallelic loss of function mutations; pNull – probability that a gene tolerates biallelic LoF, according to http://exac.broadinstitute.org. #The following programmes were used for pathogenicity prediction: DANN, MetaSVM, Polyphen2, MutationAssessor, LRTPolyphen2, MetaLR, SIFT, FATHMM, MutationTaster. All listed variants were heterozygous
